# A possible role for AMP-activated protein kinase activated by metformin and AICAR in human granulosa cells

**DOI:** 10.1186/s12958-015-0023-2

**Published:** 2015-04-10

**Authors:** Yufuko Kai, Yasushi Kawano, Hanae Yamamoto, Hisashi Narahara

**Affiliations:** Department of Obstetrics and Gynecology, Faculty of Medicine, Oita University, 1-1 Idaigaoka, Hasama, Yufu, Oita 879-5593 Japan

**Keywords:** PCOS, Ovarian function, Metformin, AMPK, Granulosa cells, Chemokine

## Abstract

**Background:**

Women with polycystic ovary syndrome (PCOS) are generally insulin- resistant and are consequently often treated with metformin. We investigated the effect of metformin and AICAR on the AMP-activated protein kinase (AMPK) pathway.

**Methods:**

We evaluated the effects of 5-amino-imidazole-4-carboxyamide-1- beta-D-ribofuranoside (AICAR) and metformin on tumor necrosis factor (TNF)-alpha- stimulated chemokine production in human granulosa cells. The phosphorylations of AMPK, I-kappaB, 4E-BP-1, p70S6K were analyzed by western immunoblotting.

**Results:**

AICAR and metformin markedly reduced the IL-8 and GROalpha production induced by TNF-alpha. AICAR and metformin also reduced the TNF-alpha-induced phosphorylation of I-kappaB. The phosphorylations of I-kappaB, 4EBP-1, p70S6K were inhibited via an AMPK-dependent signal transduction.

**Conclusions:**

These results suggest that metformin promotes granulosa cell function by reducing a TNF-alpha- and chemokine-mediated inflammatory reaction through an AMPK-dependent pathway. These finding may have implications for metformin’s actions during the treatment of PCOS with metformin.

## Background

The ovarian cycle is characterized by repeating patterns of cellular proliferation and differentiation that accompany follicular development as well as ovulation under the appropriate gonadotropin stimulation. Follicular development begins when the granulosa cells start to proliferate.

It was demonstrated that inflammatory reaction based on gene regulation is observed in polycystic ovary syndrome (PCOS) [1]. It was also reported that gene variants in several proinflammatory cytokines and their receptors, which have been recognized to be related with insulin resistance, obesity, and diabetes were found in PCOS [2]. In addition, results in gene variants of tumor necrosis factor (TNF)-α [2], type 2 TNF receptor [3], and IL-6 [4] provided supportive evidence that these factors were associated with PCOS in European populations.

It has been demonstrated that not all PCOS women are generally insulin-resistant, and PCOS is often treated with the oral biguanide metformin [5]. The systemic antihyperglycemic and insulin-sensitizing effects of metformin are well recognized [6]. However, the results of metformin treatment for women with PCOS have been variable, with some studies demonstrating induction of regular menstrual cycles and increases in both ovulation and live birth rates [7,8], versus other studies that showed no significant effect on ovulation [9-11].

Metformin is an antihyperglycemic medication that was first approved for use in 1995 and has since become a mainstay in the treatment of type 2 diabetes [11]. Metformin has also proven to be useful in the treatment of PCOS. It was shown to induce the regularity of menstrual cycles, improve hyperinsulinemia, and attenuate hyperandrogenemia in clinical studies of women with PCOS [12,13]. The exact mechanism by which metformin improves ovarian function remains unclear.

It has been reported that metformin and AICAR are known as a pharmacological activator of a adenosine monophosphate (AMP)-activated protein kinase (AMPK) [14]. AMPK is an energy-sensing enzyme with heterotrimeric complex that is believed to be directly implicated in the regulation of energy metabolism at the intracellular and whole-organ levels [15-17]. It has been reported that both the energy producing pathways and the down regulation of energy-consuming metabolic processes are activated by AMPK [15,16]. However, the possible role of AMPK is not clarified in granulosa cells.

Chemokines are recognized as a large superfamily of structurally and functionally related molecules with chemotactic activity targeted at specific leukocyte populations. They are 70–90 amino acids in length and are divided into four subfamilies based on the relative position of their cysteine residues (CC, CXC, C, CXC3). The CXC chemokine subfamily includes interleukin (IL)-8 and growth-regulated oncogene (GRO) α, GROβ, GROγ, all of which have been shown to chemoattract and activate neutrophils [18]. IL-8, a potent neutrophil chemoattract and activating factor, has been suggested to be one of the most likely agents responsible for the recruitment and activation of neutrophils in and around pre-ovulatory follicles just before ovulation [19]. Another candidate, reported to have ten times the potency of IL-8, as a neutrophil chemoattractant, is the pro-inflammatory CXC chemokine GROα [20]. It has been suggested that leukocytes may play an important role in ovulation, luteinization, and luteolysis, as they have the capacity to secrete cytokines, eicosanoids, vasoactive amines, and tissue remodeling enzymes. Leukocyte attractant activity has been detected in ovulation; however, it has not yet been fully characterized in human follicular fluids (FFs) [21]. It has been reported that IL-8 and GROα were increased by TNF-α [21]. The excess inflammatory change is thought to impair the intrafollicular circumstances.

In the present study, to examine the direct effect of metformin and AICAR on granulosa cell function, we cultured a human granulosa-like tumor cell line and investigated the regulation of TNF-α-induced chemokine production involving AMPK activation.

## Methods

### Reagents

Ham F-12 cell culture medium was purchased from Gibco-BRL (Gaithersburg, MD, USA), and Dulbecco’s minimal essential medium (DMEM) was purchased from Nissui Pharmaceutical Co. (Tokyo). Fetal calf serum (FCS) was obtained from HyClone (Logan, UT). TNF-α was obtained from R&D Systems (Minneapolis, MN). The AMP analog 5-amino-imidazole-4-carboxyamide-1-β-D-ribofuranoside (AICAR) was purchased from Sigma Chemicals (St. Louis, MO) and the metformin was from Wako (Tokyo).

### Cell culture

We used an immortalized granulosa cell line (KGN) that was established at the Kyushu University School of Medicine as described [22]. KGN cells were cultured in an equal volume of Ham F-12 and DMEM (1:1, v/v) supplemented with 10% heat-inactivated FCS with penicillin (100 IU/mL; Gibco-BRL) and streptomycin (100 mg/mL; Gibco-BRL). Cells were plated in culture dishes (12-well plates) and allowed to replicate to confluence.

The cells were then placed in the same serum-free medium for 24 h before the stimulation with TNF-α, AICAR, and metformin was initiated. After the desired length of stimulation with various concentrations of TNF-α (0.01 to 10 nM), AICAR (0.1 to 3 mM) and metformin (0.001 to 1 mM), the culture media were collected and stored at −80°C for the quantification of chemokines.

### Measurement of IL-8 and GROα

To investigate the production of IL-8 and GROα by KGN, we plated 5 × 10^5^ viable KGN cells on six-well culture plates (Corning) in 1 mL of culture medium with 10% FCS and cultured them until they were fully confluent. The cells were washed twice with phosphate-buffered saline (PBS) without calcium and magnesium, and then added to serum-free medium. The supernatant was replaced with fresh culture medium containing various concentrations of TNF-α, AICAR and metformin for 24 h. Control cells received an equivalent volume of medium alone during the incubations.

AICAR has been used as a pharmacological activator of AMPK. It can enter cells. AICAR is thought to be converted into 5-aminoimidazole-4-carboxamide ribotide (ZMP) by adenosine kinase. ZMP can activate the AMPK signaling pathway according to its structural similarity with AMP [23,24].

At the end of the culture period, the medium was stored at −80°C until assayed. These experiments were performed in triplicate and repeated four times. A commercially available enzyme-linked immunosorbent assay (ELISA; R&D Systems) was used to determine the amounts of IL-8 and GROα (R&D Systems) in the supernatants. The sensitivities of the assays for IL-8 and GROα were 0.7 pg/mL and 4.4 pg/mL, respectively. The inter- and intra-assay coefficients of variance for the ELISA of IL-8 and GROα were 10.8 and 8.2%, respectively. These experiments were performed in triplicate and repeated three times.

#### Protein preparation of KGN and Western immunoblotting analysis (ECL-WB)

To investigate the intracellular signal transduction system in KGN cells, we performed a Western immunoblotting analysis as described [25]. Briefly, 1 × 10^6^ cells were plated on a 100 mm dish (Nalgene Nunc, Rochester, NY) in 10 mL of culture medium with 10% FCS and cultured until they were fully confluent. The supernatant was replaced with fresh culture medium containing TNF-α, AICAR and metformin. Cells were stimulated by various concentrations of TNF-α (1 nM), AICAR (1 mM) and metformin (0.01 to 1 mM) for 5 min to 24 h. At the end of the culture period, the cells were washed twice with cold PBS without calcium or magnesium, harvested, pelleted, and lysed in ice-cold buffer containing 10 mM HEPES (pH 7.9), 10 mM KCl, 0.1 mM ethylenediaminetetraacetic acid (EDTA; pH 8.0), 0.1 mM ethylene glycol tetraacetic acid (EGTA), 1 mM dithiothreitol (DTT), 0.5 mM phenylmethanesulfonyl fluoride (PMSF), and 0.3 μg/mL leupeptin. The cell lysate was centrifuged for 10 min at 3,000 × g in order to pellet the nuclei. The supernatant fractions were collected and centrifuged for 10 min at 10,000 × g. The protein content was determined using a microbicinchoninic acid assay (Pierce, Rockford, IL) using bovine serum albumin (BSA) as a standard.

The lysate was mixed with loading buffer [200 mM Tris–HCl (pH 7.9), 7% sodium dodecyl sulfate (SDS; w/v), 30% glycerol (v/v), 15% 2-mercaptoethanol (v/v), and 0.75% bromophenol blue (w/v)] and heated at 95°C for 10 min. In each sample, 10 μg of protein was applied per lane. The blotted membranes were blocked in PBS containing 5% skim milk (Difco, Detroit, MI) for 1 h at room temperature and washed with three changes of Tris-buffered saline (TBS; 20 mM Tris, 137 mM NaCl, pH 7.6) buffer containing 0.1% Tween 20 for 15 min at RT. The blotted membranes were then incubated and reacted overnight with 1:1,000-diluted primary antibody [human phospho-AMPK antibody, human phospho-4EBP-1, human phospho-p70S6 kinase, rabbit polyclonal immunoglobulin G, human phospho-IκB-α, mouse monoclonal immunoglobulin G (IgG; Cell Signaling Technology), and human glyceraldehyde-3- phosphate dehydrogenase (GAPDH) antibody, mouse monoclonal immunoglobulin G (IgG, Ambion Austin, TX)] in TBS containing 5% BSA at 4°C. After being washed with three changes of TBS containing 0.1% Tween 20, the blotted membranes were incubated and reacted with 1:2,000-diluted peroxidase-conjugated secondary antibody (anti-rabbit or anti-mouse immunoglobulin γ or μ chain, goat polyclonal immunoglobulin G; Jackson Immunoresearch Laboratories, West Grove, PA) in TBS containing 5% BSA for 1 h at RT. After the membranes were washed with four changes of TBS containing 0.1% Tween 20, Lumi GLO from a Phototope-HRP Western Detection Kit (GE Healthcare UK, Buckinghamshire, England) was added to the blotted membranes and reacted for 1 min. The membranes were then covered with plastic wrap and exposed to X-ray film (GE Healthcare UK) for 1 to 2 min. These experiments were performed in triplicate and repeated three times.

### Statistical analysis

Data are presented as means ± SD and were analyzed using the Bonferroni/Dunn’s test employing StatView 4.5 software (Abacus Concepts, Berkeley, CA). P-values <0.05 were considered significant. The confidence intervals with P-values for multiple statistical analyses are at the 95% level.

## Results

### The production of IL-8 and GROα following stimulation by TNF-α in KGN cells

**Figure 1:**
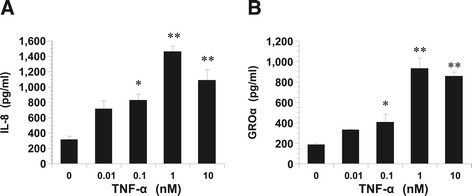
**Dose-dependent productions of IL-8 and GRO**α **stimulated with TNF-** **α** **.** Dose-dependent productions of IL-8 **(A)** and GROα **(B)** in KGN cells stimulated with TNF-α. Cultured cells were incubated with the indicated concentrations of TNF-α for 24 h at 37°C in 5% CO2. At the end of the incubation period, the conditioned medium was collected and assayed for IL-8 and GROα concentrations by ELISA. The values represent the relative ratios of IL-8 and GROα concentrations, compared with those in untreated cells after normalization with total protein. Values are the mean ± SD of the combined data of four separate experiments using different KGN preparations. **A**: *P < 0.01, **P < 0.001 (all vs.control). **B**: *P < 0.01; ** P < 0.001 (all vs. control).

We examined the effect of the addition of TNF-α at different concentrations (0.01 to 10 nM), for 24 h on the release of IL-8 by KGN. As illustrated in Figure 1, TNF-α at 0.1 nM and more caused an increase in IL-8 and GROα release.

Cell counts and 5-bromo-2′- deoxyuridine uptake at 24 h were substantially the same whether or not the cells were treated with TNF-α (data not shown). During this period, the concentrations of IL-8 and GROα in the media were significantly less in the control cells compared to the TNF-α-treated cells.

#### AICAR and metformin decreased the IL-8 and GROα production stimulated by TNF-α in KGN

**Figure 2:**
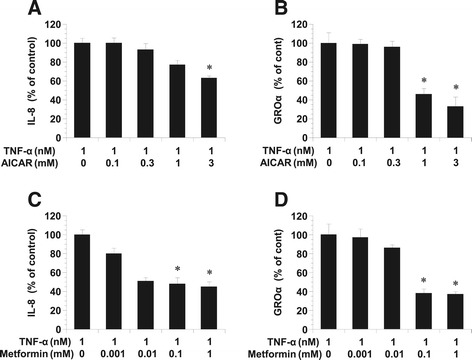
**Effects of AICAR and metformin on the TNF-**α**-induced production of IL-8 and GROα.** Effects of AICAR **(A)** and metformin **(B)** on the TNF-α-induced production of IL-8 and GROα in KGN. Cultured KGN were incubated with TNF-α and/or AICAR for 24 h at 37°C in 5% CO2. At the end of the incubation period, the conditioned medium was collected and assayed for IL-8 and GROα concentrations by ELISA. The values represent the relative ratios of IL-8 and GROα concentrations, compared with those in control cells. Values are the mean ± SD of the combined data of four separate experiments using different KGN preparations. **A**, **B**: *P < 0.001 between TNF-α vs. TNF-α + AICAR (1 mM) or TNF-α + AICAR (3 mM). **C**, **D**: *P < 0.001 between TNF-α vs. TNF-α + Metformin (0.1 mM) or TNF-α + metformin (1 mM).

We chose the KGN cell line to investigate whether AICAR could prevent inflammatory mediator production, given its role in the regulation of granulosa cells. It has been widely reported that increased IL-8 and GROα productions are associated with inflammatory reactions. KGN cultures established from human granulosa cell tumor were treated with various concentrations of AICAR or metformin prior to the addition of TNF-α. The results showed that AICAR and metformin suppressed IL-8 and GROα production in TNF-α-stimulated KGN cells in a concentration-dependent manner (Figure 2).

#### AMPK phosphorylation following stimulation by AICAR and metformin

**Figure 3:**
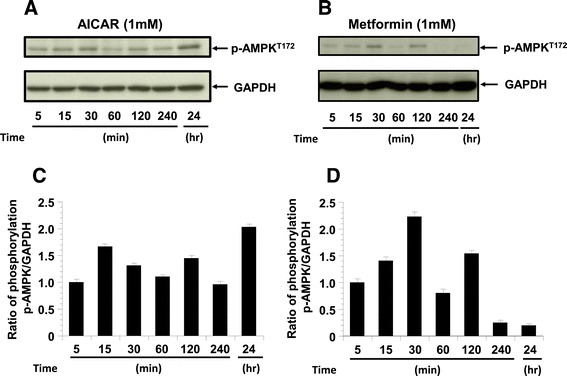
**The phosphorylation of pAMPK. The phosphorylation of pAMPK in KGN cells treated with AICAR (1 mM) or metformin (1 mM) for 5 min to 24 h (n = 4).** GAPDH is shown as the internal control. **A**: Representative blots illustrating the effect of treatment on pAMPK and GAPDH by AICAR for 5 min to 24 h. **C**: Representative blots illustrating the effect of treatment on pAMPK and GAPDH by metformin for 5 min to 24 h. The blot layout is in the same order as the graph in panels **B** and **D**, respectively.

To elucidate the role of AMPK in the KGN, we examined the effects of AICAR and metformin on the phosphorylation of AMPK. We found that 1 mM AICAR and 1 mM metformin activated AMPK time-dependently compared to the controls (5 min stimulation) (Figure 3).

#### AICAR and metformin inhibited IκB phosphorylation following stimulation by TNF-α in KGN cells

**Figure 4:**
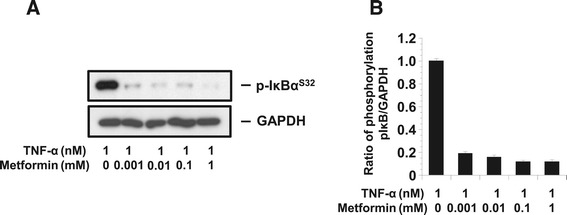
**The phosphorylation of pI** **κ** **B.** The phosphorylation of pIκB in KGN cells treated with TNF-α (1nM) and metformin (1 mM) (n = 4). **A**: The phosphorylation of pIκB was quantified against GAPDH. **B**: Representative blots illustrating the effect of treatment on pIκB and GAPDH by metformin for 15 min. The blot layout is in the same order as the graph in **B**.

To elucidate the mechanism of the TNF-α-induced secretions of IL-8 and GROα by KGN cells, we examined the effects of a TNF-α-specific mechanism, the phosphorylation of IκB. The results of the western blot analysis demonstrated that IκB phosphorylation in KGN was stimulated by TNF-α (1 nM) (Figure 4).

The anti-inflammatory effect of metformin was also apparent when cells were pretreated with metformin for only 0.5 h and then stimulated for 15 min with TNF-α. As shown in Figure 4, treatment with the combination of TNF-α and metformin resulted in reductions in the cellular protein levels of IκB phosphorylation compared to treatment with TNF-α alone. The expected 37 kDa band corresponding to GAPDH is shown as the internal control.

#### AICAR and metformin activated AMPK and inhibited translocation-related proteins in KGN cells

**Figure 5:**
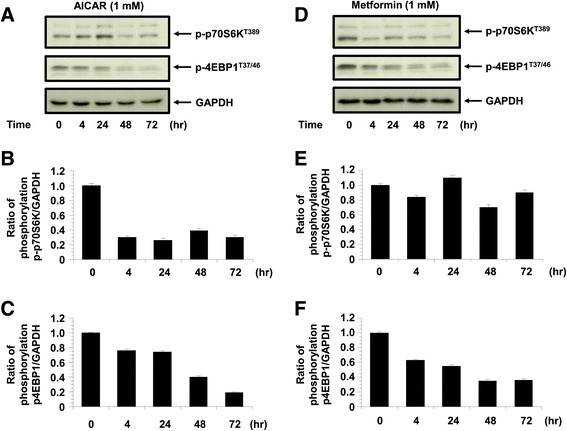
**The phosphorylation of p4E-BP1 and p70S6kinase.** The phosphorylation of p4E-BP1 and p70S6kinase in KGN cells treated with AICAR (1 mM) and metformin (1 mM) (n = 4). The phosphorylation of p4E-BP1 and p70S6kinase was quantified against GAPDH. **A**: Representative blots illustrating the effect of treatment on pAMPK and GAPDH by AICAR (1 mM) for 4 h to 72 h (n = 4). The blot layout is in the same order as the graph in panels **B** (p70S6K) and **C** (4E-BP1), respectively. **D**: Representative blots illustrating the effect of treatment on pAMPK and GAPDH by metformin (1 mM) for 4 h to 72 h (n = 4). The blot layout is in the same order as the graph in panels **E** (p70S6K) and **F** (4E-BP1), respectively.

We next examined the effects of AICAR and metformin on mTORC1 signaling, which is negatively regulated by AMPK and a major regulator of translation initiation. We assessed the phosphorylation status of its two direct downstream targets as the mTORC1 readout, p70 S6 kinase (T389) and the eukaryotic translation initiation factor 4E (eIF4E)-binding protein 1 (4E-BP1) (T37/46). AICAR time-dependently decreased the phosphorylation of p70S6K (T389), however metformin does not show significant decrease the phosphorylation of p70S6K (T389) compared to AICAR. AICAR and metformin treatment also decreased the 4E-BP1 phosphorylation, which was detected by anti phospho-4E-BP1 (T37/46) antibody (Figure 5).

## Discussion

Several cytokines, growth factors, and related proteins have been identified in human follicular fluid and in the ovary [26-28]. These molecules may exert local effects upon steroidogenesis, granulosa cell proliferation, follicular growth, and gonadotropin receptor concentration. In a previous study, Espey suggested that the ovulatory process involves inflammatory changes [29].

It has been recognized that cells usually control their growth and proliferation through a complex of intracellular signaling networks that integrate the environmental signals, energy sources, and availability of nutrients. Consequently, the balance of the signaling activities of the components of intracellular networks is necessary for proper development.

AMPK is a heterotrimeric protein that responds to increases in the ratio of adenosine-5′-monophosphate (AMP)-ATP by inducing catabolic processes that produce ATP, and by repressing energy-consuming anabolic mechanisms including protein synthesis [30,31]. AICAR has been reportedly used as a pharmacological activator of AMPK in several reports [23,24].

The AMPK-mediated suppression of protein synthesis including peptide hormones, growth factors, cytokines and chemokines results in the inhibition of substances contributing to cell growth.

In the present study, we demonstrated that AMPK, which was activated by AICAR or metformin, suppressed the TNF-α-induced IL-8 and GROα production via the inhibition of an intracellular signal transduction system in KGN cells. It was reported that AMPK suppressed the TNFα-induced cytokine production in other cells [32]. They suggest that AMPK activation attenuates the cytokine-induced expression of proinflammatory and adhesion molecule genes by inhibiting NF-κB activation [32]. Metformin, which belong to biguanide family, also inhibits the expression of proinflammatory chemokines by blocking phosphorylation and subsequent degradation of IκB-α (Figure 4). These data suggest that AICAR or metformin might suppress TNF-α-induced NFκB activation by IκB phosphorylation.

We also observed that the phosphorylation of IκB stimulated by TNF-α was reduced by AICAR- or metformin-induced AMPK activation. NFκB what dissociated from IκB and subsequently shepherded to the nucleus, was also thought to be suppressed by AICAR and by metformin. On the basis of present observations, IL-8 and GROα in FF are thought to lead to neutrophil chemoattraction and accumulation. As our results also indicated that IL-8 and GROα are regulated by a mechanism involving TNF-α, it is likely that IL-8 and GROα may play an important role in the accumulation of neutrophils and in the subsequent induction of ovulation [33]. It has been suggested that IL-8 and GROα may be important modulators of preovulatory events, not only by attracting and activating neutrophils that will eventually play a role in timely follicular rupture, but also by stimulating new blood vessel formation for the corpus luteum [19]. On the other hand, the elevation of not only white blood count and neutrophil count but also TNF-α, IL-6, CRP in serum levels have been implicated in pathphysiological findings of PCOS compared with age- and /body mass index- matched controls [34-36].

It has been reported that both mammalian targets of rapamycin (mTOR) complex (i.e., mTORC1 and mTORC2) modulate multiple cellular functions [37,38]. It was also recognized that mTORC1 regulates the activity of the eukaryotic initiation factor 4E-BP1 and the serine/threonine kinase ribosomal protein p70S6K, via interactions between these proteins and Raptor. When 4E-BP1 is hypophosphorylated, it can block protein translation by binding to eukaryotic translation initiation factor 4 epsilon (eIF4E) through eIF4 gamma (eIF4G), a protein that leads mRNA to the ribosome [39]. mTORC1 phosphorylation of 4E-BP1 leads to the dissociation of 4E-BP1 from eIF4E, allowing eIF4G to the beginning of mRNA translation [40]. In the present study, we found that the phosphorylations of 4EBP-1 and p70S6K which coexists with the downstream, were also attenuated by AMPK activation. These findings provide evidence that AMPK may contribute to both of transcription and translation in human granulosa cells.

Our study may delineate that elevation of proinflammatory cytokines such as TNF-α in women with PCOS contribute to the overproduction of IL-8 and GROα leading to the exacerbation of intraovarian circumstance. Metformin, which is administered to PCOS patients with insulin resistance, can influence not only the systemic regulation of hyperinsulinemia but also the modulation of intracellular molecules in the granulosa cells. Moreover, Gleicher et al. reported that the cause of PCOS has similarity to autoimmune disease, because active antibodies to steroidogenic enzymes in adrenal and ovary have been investigated [41]. Metformin may contribute to suppress these inflammatory reaction. A limitation of our study is that we used not primary cultured granulosa cells but a tumor cell line, because more number of cells with fixed condition for protein analysis is needed in this study.

This is a first report that metformin could affect TNF-α-induced IL-8 and GROα production and the molecular levels of signal transduction pathways in granulosa cells, which may contribute to improve intraovarian circumstance in women with PCOS patients. It was reported that the beneficial effects of metformin as a reduction of inflammatory reaction was admitted in Diabetes Mellitus. These effects are direct and independent on glucose levels [42]. Further investigations are necessary to elucidate the mechanisms of metformin’s actions. Taken together, our results demonstrate a new role for the molecular mechanism of metformin in the improvement of reproductive function in PCOS.

## Abbreviations

AICAR: 5-amino-imidazole-4-carboxyamide-1- β-D-ribofuranoside
AMPK: Adenosine monophosphate-activated protein kinase
DTT: Dithiothreitol
EDTA: Ethylenediaminetetraacetic acid
EGTA: Ethylene glycol tetraacetic acid
GROα: Growth-regulated oncogene α
IL-6: Interleukin-6
IL-8: Interleukin-8
NFκB: Nuclear factor-κB
PCOS: Polycystic ovary syndrome
PMSF: Phenylmethanesulfonyl fluoride
TNF-α: Tumor necrosis factor-α
